# Regional Distribution and Evolution of Gray Matter Damage in Different Populations of Multiple Sclerosis Patients

**DOI:** 10.1371/journal.pone.0135428

**Published:** 2015-08-12

**Authors:** Massimiliano Calabrese, Richard Reynolds, Roberta Magliozzi, Marco Castellaro, Aldo Morra, Antonio Scalfari, Gabriele Farina, Chiara Romualdi, Alberto Gajofatto, Marco Pitteri, Maria Donata Benedetti, Salvatore Monaco

**Affiliations:** 1 Neurology Section, Department of Neurological and Movement Sciences, University of Verona, Verona, Italy; 2 Neuroimaging Unit, Euganea Medica, Padova, Italy; 3 Division of Brain Sciences, Faculty of Medicine, Imperial College London, Hammersmith Hospital, London, United Kingdom; 4 Department of Cell Biology and Neuroscience, Istituto Superiore di Sanità, Rome, Italy; 5 Department of Information Engineering, University of Padova, Padova, Italy; 6 Department of Medicine, Division of Brain Sciences, Centre for Neuroscience, Wolfson Neuroscience Laboratories, Imperial College London, London, United Kingdom; 7 Department of Clinical and Experimental Medicine, University of Sassari, Sassari, Italy; 8 Department of Biology, University of Padova, Padova, Italy; University of Jaén, SPAIN

## Abstract

**Background:**

Both gray-matter (GM) atrophy and lesions occur from the earliest stages of Multiple Sclerosis (MS) and are one of the major determinants of long-term clinical outcomes. Nevertheless, the relationship between focal and diffuse GM damage has not been clarified yet. Here we investigate the regional distribution and temporal evolution of cortical thinning and how it is influenced by the local appearance of new GM lesions at different stages of the disease in different populations of MS patients.

**Methods:**

We studied twenty MS patients with clinically isolated syndrome (CIS), 27 with early relapsing-remitting MS (RRMS, disease duration <5 years), 29 with late RRMS (disease duration ≥ 5 years) and 20 with secondary-progressive MS (SPMS). The distribution and evolution of regional cortical thickness and GM lesions were assessed during 5-year follow-up.

**Results:**

The results showed that new lesions appeared more frequently in hippocampus and parahippocampal gyri (9.1%), insula (8.9%), cingulate cortex (8.3%), superior frontal gyrus (8.1%), and cerebellum (6.5%). The aforementioned regions showed the greatest reduction in thickness/volume, although (several) differences were observed across subgroups. The correlation between the appearance of new cortical lesions and cortical thinning was stronger in CIS (r^2^ = 50.0, p<0.001) and in early RRMS (r^2^ = 52.3, p<0.001), compared to late RRMS (r^2^ = 25.5, p<0.001) and SPMS (r^2^ = 6.3, p = 0.133).

**Conclusions:**

We conclude that GM atrophy and lesions appear to be different signatures of cortical disease in MS having in common overlapping spatio-temporal distribution patterns. However, the correlation between focal and diffuse damage is only moderate and more evident in the early phase of the disease.

## Introduction

Multiple Sclerosis (MS) is an autoimmune [1], chronic and disabling disease of the human central nervous system, characterized histologically by multifocal areas of inflammatory demyelination within the white matter (WM) [2], accompanied by varying degrees of axonal loss [3]. Nevertheless, several pathologic and MRI studies have suggested that extensive cortical and deep gray matter (GM) atrophy occurs from the earliest stages of the disease [4], being one of the major determinants of long-term clinical outcomes in MS [5,6]. Indeed, physical and cognitive disability seems to correlate better with GM damage rather than with WM lesion load [6,7].

Understanding the mechanisms underlying cortical atrophy is challenging [8]. GM and WM damage appear to be at least partly independent, albeit simultaneous components of the disease, and only a weak relationship has been obtained between WM lesion load and cortical lesions [9], or cortical atrophy [10,11]. Conversely, several MRI studies suggested that cortical thinning [12] and cortical lesions [13] can be present even at the clinical onset of the disease and in a primary progressive subset [11], in association with a low WM lesion load. In the light of these data, it is unlikely that regional changes in cortical volume are primarily the consequence, via retrograde degeneration, of ongoing axonal transection in subcortical WM lesions. On the contrary, GM damage might result from a more diffuse inflammatory process directly targeting the GM itself [14,15]. Observations on GM lesions in post-mortem MS brain tissues of patients with progressive disease have demonstrated a lower extent of lymphocyte and macrophage infiltration compared to WM lesions [15,16]. However, the presence of both diffuse and lymphoid-like immune cell infiltrates in the meninges of patients with secondary progressive (SP) MS was recently found to be associated with increased subpial demyelination, loss of neurons and their extensions, and a more severe disease course [17]. In line with these data, a recent study on a large number of brain biopsies from patients with early MS showed a close association between actively demyelinating CLs and meningeal inflammation [18]. In addition, data from natural history studies suggest that the outcome severity is largely determined during the initial clinical phase, highlighting the importance of early pathological changes as determinants of the long-term prognosis [19,20].

Although a correlation between CL load and the severity of GM atrophy was previously found [6], a conclusive proof of a cause-effect relationship between the appearance of CLs and the development of cortical atrophy is still lacking.

In this context, we set out to investigate longitudinally, and at different disease stages, the regional distribution and temporal evolution of cortical lesions and cortical thinning in MS patients. In addition, we explored whether the local appearance of new CLs may influence the development of cortical atrophy in the same region.

## Materials and Methods

### Study population

Ninety-six consecutive patients, currently followed at the Multiple Sclerosis Centre of the Neurology Section, University Hospital of Verona (Verona, Italy), and having at least 5-year of longitudinal MRI follow up performed with the same MRI scan and the same MRI protocol at the Neuroradiology Unit of Euganea Medica (Padova, Italy), have been included in this retrospective study (Table 1).

**Table 1:** Demographical, clinical, and MRI characteristics of the studied population.

	CIS (n = 20)	Early RRMS (n = 27)	Late RRMS (n = 29)	SPMS (n = 20)
Gender (F; M)	13; 7	19; 8	19; 10	15; 5
Age (years)	30.1±9.8; 18–51	31.4±10.0; 18–48	32.8±7.7; 19–55	43.1±8.4; 33–59
Disease duration (years)	0.4±0.1; 0–0.8	3.2±0.9; 1–4	8.6±2.3; 6–13	16.8±5.9; 10–22
EDSS	1.0±0.6; 0–2.0	1.6±0.6; 1–3.5	2.5±0.9; 1.5–4.5	4.5±1.2; 3.0–7.0
T2 WM lesion load (cm^3^)	1.3±1.0; 0.4–3.9	7.4±5.4; 1.6–19.2	9.5±5.4; 1.8–23.9	16.6±10.3; 4.7–45.9
Global CTh (mm)	2.50±0.21; 2.01–2.93	2.42±0.18; 1.88–2.89	2.28±0.10; 1.68–2.6	2.15±0.20; 1.75–2.61
CLs number	1.1±0.7; 0–4	2.3±1.0; 0–6	3.9±1.6; 0–12	6.9±1.7; 2–28

F = female, M = male; WM = white matter; CTh = cortical thickness; CLs = cortical lesions.

At the beginning of the follow-up (onset, T0), according to the MS diagnostic criteria [21], 20 patients were considered as having clinically isolated syndrome (CIS), 27 early Relapsing Remitting MS (RRMS, disease duration < 5 years), 29 late RRMS (disease duration ≥ 5 years), and 20 Secondary Progressive MS (SPMS). Table 1 shows demographic and clinical characteristics of the studied population at onset; 21 early RRMS, 22 late RRMS and 2 SPMS were treated with IFN beta 1a, IFN beta 1b or Glatiramer Acetate, 12 SPMS were treated with Cyclophosphamide and 4 RRMS (3 early and 1 late RRMS) were treated with Natalizumab.

By the end of the follow-up 5 years later (endstate, T5), 13 CIS had a transition to early RRMS, while 3 late RRMS entered the SP phase. Nevertheless, during the data analysis each patient has been considered belonging to his/her original group despite the switching to one of the other groups. Fifty-one patients (9 CIS, 41 RRMS, and 1 SPMS) had at least 1 relapse during the observation period: among these, 32 (4 CIS, 27 RRMS, and 1 SPMS) had an increase in the EDSS score (median 1.0; range 0.3) related to the relapse and confirmed at 6 months after the relapse. Sixty-four RRMS were treated with IFN beta 1a, IFN beta 1b or Glatiramer Acetate, 6 RRMS were treated with Natalizumab and 4 with Fingolimod, 3 SPMS were treated with Cyclophosphamide and 4 SPM±S were treated with Azathioprine while the remaining 10 patients were untreated.

The Ethic Committee of the University Hospital of Verona (Verona, Italy) approved the study and written informed consent was obtained from all patients before the data analysis.

### Image acquisition protocol

Each patient underwent the same MR protocol at T0 and at T5 (range = 62 ± 2 months). All images were acquired at the Neuroradiology Unit of Euganea Medica (Padova, Italy), using the same 1.5 T Philips Achieva scanner with 33 mT/m power gradient, and a 16-channel head coil. No major hardware upgrades of the scanner occurred during the study period. The following images were acquired from each subject: 1) *3D Double Inversion Recovery (DIR)*: 3D sequence without any interpolation techniques, repetition time (TR) 6.500 msec, inversion time 2.800 msec, delay 500 ms, echo time (TE) 265 msec, slice thickness 1.5 mm, number of averages 2, matrix 256 x 256); 2) *3D Fluid-Attenuated Inversion Recovery (3D FLAIR)*: TR = 10000 msec, TE = 120 msec, TI = 2500 msec, ETL = 23, slice thickness = 1.5 mm, a matrix size = 172 x 288, and a FOV = 250 x 200 mm^2^; 3) *Three volumetric fast-field echo sequence*: 120 contiguous axial slices, TR = 25 msec, TE = 4.6 msec, flip angle = 30°, slice thickness = 1.0 mm, matrix size = 256 x 256, and a FOV = 250 x 250 mm^2^ were acquired. At follow-up, subjects were carefully repositioned according to published guidelines for serial MRI studies of MS [22].

### Image analysis

All images were evaluated by a neurologist (MC) and a neuroradiologist (AM) both with large experience on neuroimaging of MS patients.

#### Regional cortical thickness/volume evaluation

Cortical reconstruction and volumetric segmentation was performed at T0 and at T5 on a volumetric T1-weighted data set by means of the *longitudinal stream* included in the *Freesurfer image analysis suite* (release v5.3.0), available online (*http*:*//surfer*.*nmr*.*mgh*.*harvard*.*edu/* *)*. The technical details of these procedures have been described previously [23]. Topological defects in cortical surfaces due to white matter hypointensities were detected and manually corrected to have an accurate cortical segmentation. Since no significant differences were observed between right and left hemisphere, we decided to average the measures from both hemispheres [4,12].

The cortical parcellation (for regional analysis) was performed on the base of the Talairach Atlas, included in Freesurfer [23].

#### Cortical and WM lesion evaluation

At T0 and T5, the number of new and pre-existing CLs was assessed region by region on DIR images by consensus following the recent recommendations for CL scoring in patients with MS [24]. Since no difference between right and left hemisphere were observed [25], an averaged measure was calculated. The same procedure was applied to FLAIR images to identify brain WM lesions, thus obtaining the number of brain WM at T0 and T5.

### Statistical analyses

Differences among MS subtypes, between patients having more or less than 5 years of disease duration, and patients developing or not new CLs during the study, were assessed through analysis of variance (ANCOVA), including treatment as covariate (this considering the possible effect of disease modifying drugs on grey matter atrophy) [26] and *post hoc* Tukey HSD procedure to account for multiple comparisons. Also differences between CTh changes in region with new CLs compared to regions without new CLs were assessed through analysis of variance (ANOVA). Since CLs were not homogeneously distributed, the Mann-Whitney test was used to compare populations with respect to their CL number. Pearson Chi Square was applied to test the difference between patients. Univariate correlation using the Pearson coefficient has been applied to test the correlation between the baseline number of CLs and the entity of the global CTh change and also between the number of new CLs and the global CTh change.

## Results and Discussion

### Spatiotemporal distribution of cortical lesions across different MS subtypes

**Fig 1:**
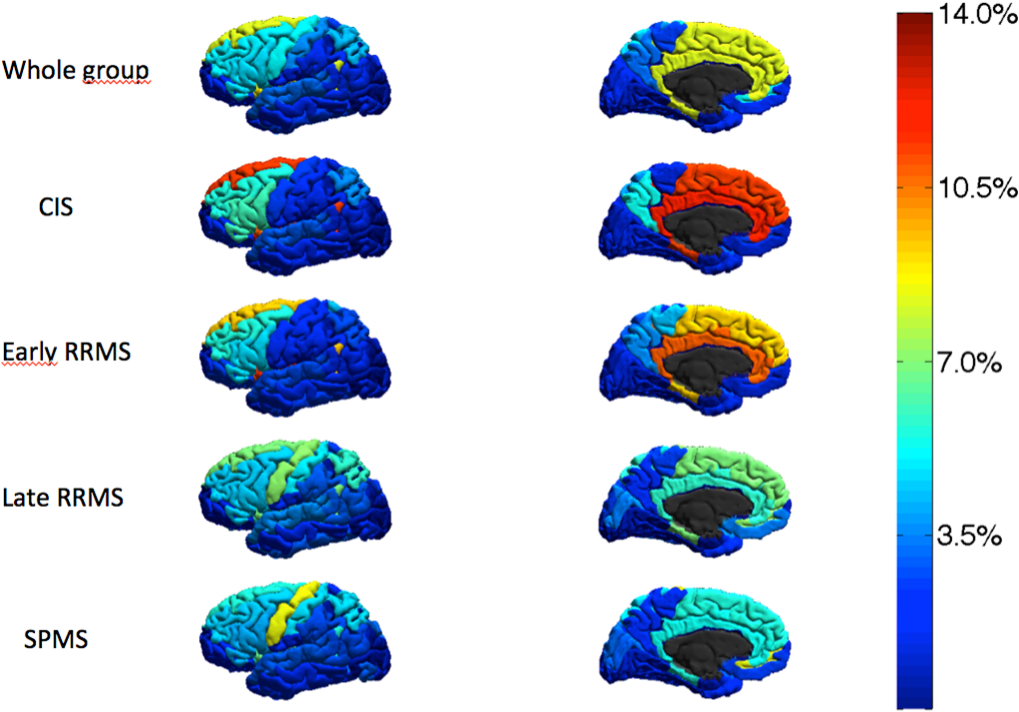
3D Regional map of the frequency of the appearance of new grey matter lesions during the 5-year follow up in the whole group and in the different MS subsets.

A minimal, anonymized dataset underlying the results of the present study is available (S1 Dataset). At baseline, in the whole group 334 CLs were identified (22 in CIS, 61 in early RRMS, 113 in late RRMS and 138 in SPMS; Table 1). The most affected areas were the cingulate cortex (9.3% ± 2.1%; range 5.6%-14.2%), the hippocampus and the parahippocampal gyrus (8.8 ± 2.6%; range 4.2%-12.4%), the insula (8.2 ± 3.2%; range 3.2%-15.2%), the superior frontal gyrus (8.1 ± 1.7%; range 5.3%-11.4%) and the cerebellum (7.9 ± 3.2%; range 4.2%-16.2%). However, the distribution was not homogeneous in all subsets of patients: in CIS and early RRMS, CLs were located more frequently in fronto-temporal regions while they were more widespread in late RRMS and SPMS (Fig 1).

After 5 years, 331 new CLs (48 in CIS group, 115 in early RRMS group 121 in late RRMS group and 47 in SPMS group) were identified (mean 3.6 ± 4.1, range = 1–18). No significant differences were observed in the number of new CLs between early RRMS (4.9 ± 1.8, range = 0–18) and late RRMS group (4.2 ± 1.9, range = 0–12) while the number of new CLs was significantly lower in SPMS group (1.4 ± 1.3, range = 0–5, *p* < 0.001) and in CIS group (2.4 ± 1.0, range = 0–6, *p* < 0.001). However, when the number of new CLs was calculated only in those CIS that converted to definite MS during the following 5 years (4.7 ± 1.3, range = 0–6), no significant difference was observed compared to RRMS group (*p* = n.s.).

New CLs appeared more frequently in the hippocampus and the parahippocampal gyrus (9.1%), the insula (8.9%), the cingulate cortex (8.3%), the superior frontal gyrus (8.1%), and the cerebellum (6.5%). Importantly, significant differences were observed between different disease subtypes (Fig 1, Table 2, and S1 Table) and according to the disease duration (S2 Table).

**Table 2:** New cortical lesions (%) and cortical thickness change (%) after 5 years of follow-up.

	Whole group (n = 96)				CIS (n = 20)				early RRMS (n = 27)				Late RRMS (n = 29)				SPMS (n = 20)			
	New CLs		CTh change		New CLs		CTh change		New CLs		CTh change		New CLs		CTh change		New CLs		CTh change	
	Mean	SD	Mean	SD	Mean	SD	Mean	SD	Mean	SD	Mean	SD	Mean	SD	Mean	SD	Mean	SD	Mean	SD
Hippocampal and parahippocampal	9,1%	3,2%	5,2%	2,8%	12,7%	2,9%	7,7%	2,0%	13,5%	2,8%	5,6%	2,2%	5,4%	2,1%	3,9%	2,0%	5,1%	2,5%	3,7%	2,1%
Insular	8,9%	3,4%	5,4%	1,8%	10,9%	2,7%	6,2%	1,9%	11,4%	3,0%	7,0%	1,8%	6,8%	2,4%	3,6%	1,4%	6,6%	2,7%	4,0%	1,8%
Cingulate	8,3%	3,6%	5,0%	2,9%	11,7%	3,2%	7,0%	2,4%	10,8%	3,5%	6,7%	2,9%	5,7%	2,3%	3,8%	2,3%	5,4%	3,6%	3,0%	2,5%
Frontal superior	8,2%	4,0%	4,5%	1,6%	11,2%	2,8%	7,2%	1,1%	9,3%	3,8%	4,3%	1,3%	6,9%	3,1%	3,8%	1,2%	5,6%	3,2%	2,8%	1,4%
Cerebellum	6,8%	1,0%	5,2%	1,5%	3,9%	0,6%	3,5%	1,2%	4,4%	0,8%	3,0%	1,1%	8,4%	2,4%	6,8%	2,1%	10,4%	0,8%	6,5%	1,0%
Precentral	5,1%	2,3%	3,9%	1,6%	2,6%	2,6%	1,3%	1,1%	2,4%	2,1%	1,0%	0,9%	7,0%	2,1%	6,9%	1,7%	8,5%	2,6%	6,1%	0,9%
Frontal middle	4,9%	3,1%	2,8%	2,0%	5,5%	2,3%	2,3%	1,7%	5,5%	2,0%	3,2%	2,0%	4,4%	1,4%	2,1%	1,6%	4,4%	2,0%	3,3%	1,6%
Frontal Inferior	4,8%	4,5%	2,0%	4,0%	6,1%	2,1%	2,9%	2,5%	4,8%	2,2%	2,0%	3,2%	4,5%	1,2%	1,8%	1,8%	4,1%	2,1%	1,1%	3,2%
Parietal superior	4,3%	3,2%	2,1%	2,2%	3,7%	3,0%	0,9%	2,1%	2,7%	3,2%	1,6%	1,9%	5,9%	1,4%	2,6%	1,9%	4,8%	3,0%	3,1%	1,9%
Postcentral	3,5%	1,2%	4,0%	2,7%	2,2%	0,7%	1,1%	2,5%	2,2%	0,9%	3,2%	2,1%	4,9%	1,6%	6,5%	2,1%	4,7%	0,9%	6,5%	2,0%
Precuneus	3,2%	1,0%	3,8%	3,4%	5,4%	1,1%	1,4%	2,3%	3,5%	1,0%	4,9%	2,3%	2,0%	0,9%	3,5%	2,3%	2,2%	0,9%	4,0%	2,3%
Temporal superior	3,1%	1,5%	4,0%	4,2%	2,9%	1,0%	3,4%	3,6%	2,7%	1,0%	3,4%	4,0%	3,6%	1,4%	4,8%	2,0%	3,0%	1,0%	4,3%	3,2%
Paracentral	2,6%	0,9%	3,5%	2,0%	1,1%	0,9%	2,1%	1,6%	4,3%	0,9%	2,6%	1,6%	2,6%	0,9%	3,2%	1,5%	2,0%	0,9%	5,4%	1,5%
Temporal inferior	2,5%	1,0%	1,6%	1,6%	2,1%	1,0%	1,2%	1,9%	2,6%	1,0%	1,5%	1,8%	2,9%	1,0%	2,0%	1,8%	2,4%	1,0%	1,6%	1,8%
Parietal inferior	2,2%	1,1%	3,5%	2,1%	1,4%	1,3%	1,7%	2,1%	1,6%	1,1%	2,4%	1,8%	2,9%	0,8%	5,3%	2,4%	2,8%	1,1%	4,4%	1,8%
Temporal middle	2,1%	0,9%	2,1%	5,0%	2,0%	0,5%	2,1%	4,1%	2,3%	0,9%	1,9%	3,9%	2,0%	0,9%	2,5%	3,6%	2,3%	0,9%	2,0%	3,6%
Cuneus	2,1%	0,4%	4,5%	3,3%	1,0%	0,3%	2,5%	3,1%	1,2%	0,4%	1,8%	3,0%	3,3%	0,4%	6,2%	2,1%	2,9%	0,4%	7,2%	2,5%
Rectus	2,1%	1,0%	2,6%	1,6%	1,0%	1,0%	1,3%	1,8%	1,0%	1,0%	1,0%	1,6%	3,4%	1,4%	3,1%	1,3%	2,6%	1,0%	4,7%	1,6%
Orbital	1,9%	1,5%	2,7%	1,9%	2,4%	1,4%	3,6%	1,2%	2,5%	1,5%	5,3%	1,1%	1,1%	1,7%	1,4%	1,1%	1,6%	1,4%	1,4%	1,1%
Occipital inferior	1,9%	0,9%	3,5%	1,8%	0,9%	0,7%	1,8%	1,5%	1,2%	0,9%	1,8%	1,3%	2,4%	0,9%	4,2%	1,1%	2,9%	0,9%	6,0%	1,1%
Occipital superior	1,8%	0,8%	3,2%	1,8%	0,8%	0,5%	2,4%	1,6%	0,8%	0,8%	1,9%	1,6%	2,6%	0,8%	4,3%	1,2%	3,2%	0,8%	4,3%	1,6%
Calcarine	1,8%	0,9%	4,9%	1,7%	1,0%	0,8%	3,1%	1,3%	1,0%	0,8%	5,2%	1,3%	2,4%	0,9%	5,1%	2,1%	2,9%	0,8%	7,3%	0,9%
Subcentral	1,8%	1,1%	3,2%	1,8%	1,6%	1,2%	2,5%	1,4%	1,3%	1,1%	2,2%	1,2%	2,2%	1,0%	3,7%	1,6%	2,0%	1,1%	4,3%	1,5%
Frontomarginal	1,6%	0,4%	3,4%	1,2%	1,4%	0,7%	2,1%	1,0%	1,7%	0,5%	4,6%	1,0%	1,6%	0,5%	3,5%	1,0%	1,6%	0,5%	3,5%	1,0%
Temporal pole	1,5%	1,1%	2,0%	1,5%	1,0%	1,0%	1,7%	1,5%	1,0%	1,0%	1,7%	1,5%	2,3%	1,0%	2,3%	1,3%	1,7%	1,0%	2,3%	1,3%
Occipital pole	1,0%	0,5%	3,0%	1,2%	0,9%	0,6%	1,0%	1,1%	1,5%	0,5%	1,0%	1,0%	0,6%	0,4%	4,1%	0,9%	1,1%	0,4%	5,5%	0,9%
Frontopolar	0,8%	0,5%	4,2%	1,3%	0,4%	0,3%	2,4%	1,6%	1,1%	0,5%	3,5%	1,2%	0,7%	0,8%	4,0%	1,8%	1,1%	0,6%	6,9%	1,2%
Occipito-temporal	0,8%	0,3%	2,6%	1,4%	1,3%	0,3%	2,2%	1,1%	0,8%	0,3%	2,5%	1,1%	0,5%	0,3%	2,9%	1,0%	0,9%	0,3%	2,9%	1,0%
Lateral fissure	0,5%	0,2%	3,3%	2,4%	0,5%	0,2%	0,9%	0,8%	0,5%	0,2%	2,5%	1,2%	0,4%	0,1%	4,1%	2,0%	0,6%	0,2%	5,2%	1,3%
Occipital middle	0,5%	0,2%	1,9%	1,3%	0,4%	0,2%	1,5%	1,0%	0,4%	0,2%	1,5%	1,0%	0,6%	0,2%	2,7%	1,0%	0,6%	0,2%	2,0%	1,0%

CLs = cortical lesions; CTh = cortical thickness; SD = standard deviation; CIS = clinically isolated syndrome.

### Spatiotemporal evolution of cortical thinning across different MS subtypes

Global CTh at T0 even after age correction, was significantly lower in SPMS (2.15 ± 0.20 mm; range = 1.75–2.61 mm) and in late RRMS (2.28 ± 0.14 mm; range = 1.68–2.66 mm) compared to early RRMS (2.42 ± 0.18 mm; range = 1.88–2.89 mm) and CIS (2.50 ± 0.21 mm; range = 2.01–2.93 mm), (SPMS vs. CIS: *p* < 0.001; SPMS vs. early RRMS: *p* = 0.002; late RRMS vs. CIS: *p* = 0.004). As expected, a moderate correlation was observed between global CTh and disease duration (r^2^ = -0.574, *p* < 0.001).

After 5 years follow-up, the mean CTh change was higher in SPMS (4.2% ± 0.9%; range = 2.7–5.8%) and in late RRMS (3.7% ± 0.7%; range = 2.3–5.9%) compared to early RRMS (3.0% ± 0.6%; range = 1.8–4.3% *p* < 0.001 vs. SPMS and *p* = 0.041 vs. late RRMS) and CIS (2.5% ± 0.8%; range = 1.7–4.4%, *p* < 0.001 vs. SPMS and late RRMS), indicating increasing loss of cortical GM volume with increasing disease duration.

**Fig 2:**
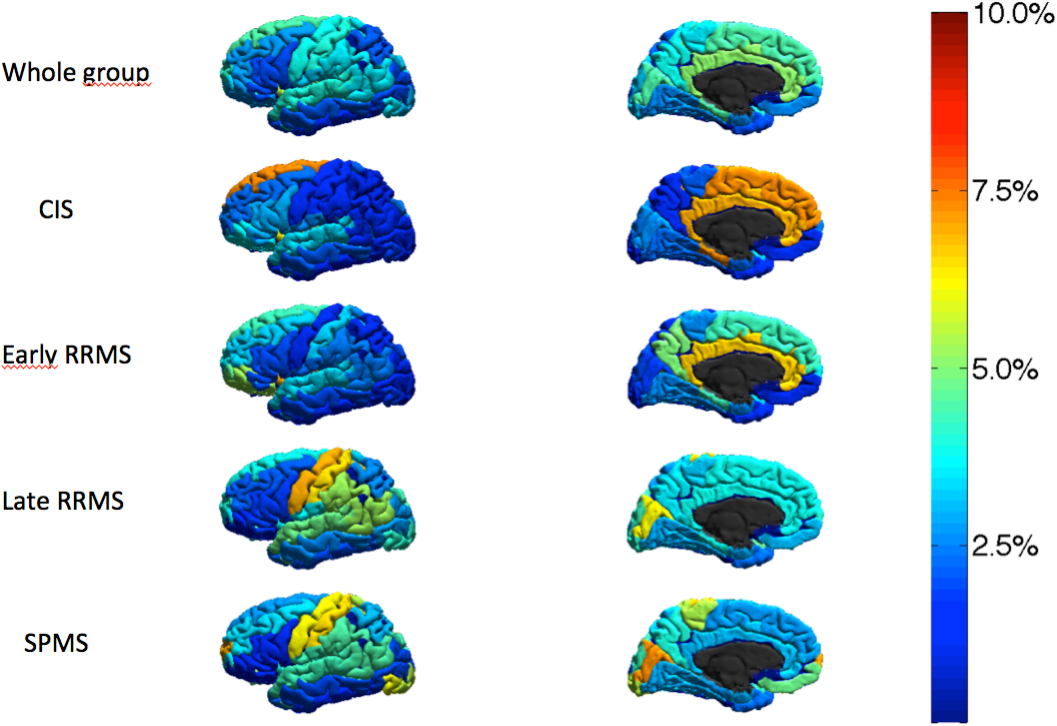
3D Regional map of the cortical thickness change during the 5-year follow up in the whole group and in the different MS subsets.

In the whole group, the regional analysis revealed that the insula (5.4%), the cerebellum (5.2%), the hippocampus and the parahippocampal gyrus (5.2%), and the cingulate cortex (5.0%) showed the greatest reduction in thickness/volume (Fig 2, Table 2, and S1 Table).

The development of regional cortical thinning was not homogeneous across different MS groups. The reduction of CTh and volume of the hippocampus and the parahippocampal gyrus, the insula, and the cingulate cortex were particularly severe in CIS and early RRMS patients whereas in late RRMS and SPMS cortical thinning and volume loss were significantly greater in the precentral gyrus, the postcentral gyrus, and the cerebellum (Fig 2, Table 2, and S1 Table).

### Relationship between CLs and CTh evolution

The mean volume of CLs at baseline moderately correlated with global CTh change (r^2^ = 0.26, *p* < 0.001) in the following 5 years; however, such correlation was stronger in CIS (r^2^ = 0.34, *p* < 0.001) and early RRMS (r^2^ = 0.38, *p* < 0.001) compared to RRMS (r^2^ = 0.16, *p* = 0.029) and SPMS (r^2^ = 0.09, *p* = 0.311).

**Fig 3:**
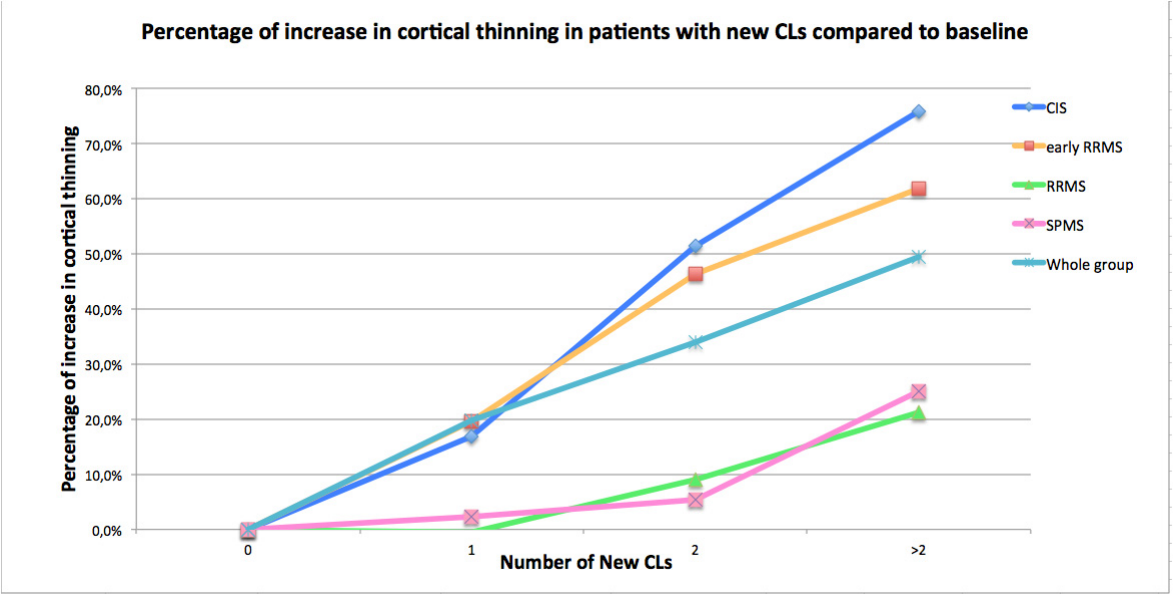
Relationship between the percentage of increase in cortical thinning and the appearance of new grey matter lesions during the 5-year follow up in the whole group and in the different MS subsets. As the image shows, patients with new cortical lesions showed higher cortical thinning; but this was more evident in CIS and early RRMS patients. The results are express as percentage of change from baseline, being the baseline the cortical thickness change when the number of new cortical lesions are 0.

Patients with the appearance of at least 2 CLs showed higher global CTh change (3.9% ± 0.6%; range = 1.7%-6.9%) compared to patients with no new CLs (2.5% ± 0.7%; range = 1.7%-4.0%, *p* < 0.001). The total number of new CLs moderately correlated with the global CTh change in the whole group (r^2^ = 0.26, *p* < 0.001). However, again, such a correlation was stronger in CIS (r^2^ = 0.50, *p* < 0.001) and in early RRMS (r^2^ = 0.52, *p* < 0.001), compared to late RRMS (r^2^ = 0.25, *p* < 0.001) and SPMS (r^2^ = 0.06, *p* = 0.133; Fig 3). On the contrary, the number of new CLs per region did not correlate with the CTh change within the same region.

Finally, a modest correlation was also observed between T2-WMLV at baseline and the CTh change (r^2^ = 0.19, *p* < 0.001), while no correlation was observed between the appearance of new WM lesions and the CTh change in the whole group nor in the 4 subtypes (*p* = n.s.).

GM damage is a relevant and early phenomenon in MS with significant impact on progression of physical and cognitive disability [5,6]. GM atrophy and lesions are two different expressions of such damage that can be monitored in vivo by MRI [9,10]. Nevertheless, the distribution and the temporal evolution of regional cortical thinning in MS, and also how it is influenced by the local appearance of new CLs, have not been clarified yet.

The current 5-year longitudinal study on different subgroups of MS patients shows that some cortical regions, such as the cingulate cortex, the hippocampus, the insula, the superior frontal gyrus, and the cerebellum are more susceptible to focal (i.e., lesions) and diffuse (i.e., thinning) damage than other regions. Our data are in line with previous MRI studies [25], including the observation of a correlation between early structural and functional changes in the hippocampus and the insula, and cognitive dysfunction [27]. The present data are also supported by robust histopathological evidence [28,29] and also by the observation that extensive lymphoid-like meningeal immune cell infiltrates, associated with increased subpial demyelination and localized to the deep sulci, were most frequently detected in the same cortical regions [16].

Taken together, these results strengthen the hypothesis that a higher susceptibility to neurodegenerative processes in key brain regions, known to be related to specific clinical (cognitive) functions, is likely to underlie the clinical manifestations of at least a subgroup of MS patients [30]. Nevertheless, the relationship between some clinical manifestations and GM damage is not exclusive since several data remarked the crucial role of WM tracts integrity, especially in cognitive deterioration [31]. As several recent studies have pointed out [32,33], it looks like that the ultimate responsible of clinical and cognitive deterioration is more a combination of a diffuse WM and GM damage (especially in specific brain areas) rather than a severe but isolated GM or WM damage.

Although understanding the origin of cortical damage in MS is still challenging, some considerations can be done on the basis of this longitudinal study.

First, we observed that the distribution of GM damage is not homogeneous across different disease subtypes and, in turn, different disease durations. Both focal and diffuse GM damage seem to affect in the earliest phases of the disease (CIS and early RRMS) the fronto-temporal regions, especially the hippocampus and the parahyppocampal gyrus, the insula and the cingulate cortex, while they become more widespread, involving also the precentral gyrus, the postcentral gyrus and the cerebellum, later in the disease course (late RRMS and SPMS).

Only in CIS and early RRMS we have found a strong correlation between the appearance of CLs and the CTh change suggesting that, at least at the beginning of the disease, the early focal cortical pathology plays a relevant role in the development of brain atrophy. This is in line with natural history studies, demonstrating that the outcome severity is primarily determined during the early phase [19,20]. The late disease evolution becomes relatively stereotyped among patients and largely uninfluenced by the early rate of disability accumulation. Taken together, these data further support the notion that pathological mechanisms, affecting the long-term prognosis, are already active during the early course of the disease.

It is worth to underline that such correlation, and even the partial overlap between focal and diffuse damage, do not imply that CLs are the main cause of cortical thinning. Indeed, the relationship between new CLs and cortical thinning does not exist at the level of single cortical areas but only, in the whole brain, between the total number of new lesions and the global cortical thickness change. This means that, at least at the beginning of the disease, those patients with the highest accumulation of new CLs showed the greatest global cortical thinning. In the advanced disease phases, it seems that other factors may influence the development of cortical atrophy as suggested by the high cortical thinning in some regions, such as the calcarine fissure, that show low frequency of CLs presence. Whether this is the consequence of tissue destruction in the subcortical WM, involving axonal transection and retrograde neurodegeneration [34], it is has not been clarified yet. However, a voxel base morphometry analysis showed that peripapillary retinal nerve fiber layer thinning was specifically associated with atrophy of the visual cortex thus suggesting that trans-synaptic degeneration might be a contributor to chronic axon damage in MS [35].

A second hypothesis is that cortical thinning in these areas might be more dependent on diffuse subpial CLs [15], which are the most frequent type of CLs seen in post-mortem MS brains, but almost invisible by MRI. We are aware that the main limitation of our study is that it was performed on a 1.5 T scanner, which even though using the DIR sequence, does not allow a clear identification of the entire cortical pathology and especially of subpial demyelination. We are also aware that it is generally accepted that 7T MRI is much better at detecting cortical lesions compared to conventional 3T MRI and 1.5T MRI [36,37]. Nevertheless, the identification of subpial demyelination is still a challenge even on a 7T MRI and a longitudinal study including high number of patients is almost unworkable at 7T MRI. Moreover, a recent histopathological study has confirmed a significant correlation between MRI visible CLs (at 1.5T) and the total amount of GM tissue damaged [38], suggesting that MRI visibility of CLs seems determined more by lesion size than by any distinctive underlying pathology.

A third hypothesis suggests that, in addition to the role of demyelination in cortical thinning, there is a diffuse loss of neurons, axons, and synapses in the non-demyelinated normal appearing gray matter [17,39,40], which might explain the more general GM atrophy not associated with lesions. This would be also in line with recent imaging studies showing several early abnormalities even in the normal appearing GM [41,42].

This retrospective study is not free from limitations, mainly related to the low MRI field applied and to the low sensitivity of DIR sequence for GM damage when compared to the neuropathological approach. Moreover, the study do not provide any MRI data about the pathology of the normal appearing WM that may significantly contribute to cortical atrophy progression in MS [43].

However, this work has also several strengths: the longitudinal approach, the high number of patients included in the analysis, and the fact that, for the first time, a comparison between the appearance of CLs and cortical thinning has been done region-by-region and in different MS populations.

Concluding, from the clinical point of view, considering the potential effect of some new disease-modifying drugs on the cerebro-spinal fluid (CSF) proteome and on the accumulation of CLs [42,44], the present results would suggest that these drugs should be used as early as possible when their effect on the accumulation of CLs might be still in time to prevent the development of cortical atrophy and consequent irreversible disability.

## Supporting Information

S1 Dataset Minimal, anonymized dataset underlying the results of the present study. (XLSX)

S1 Table New cortical lesions and cortical thickness change after 5 years follow-up. (PDF)

S2 Table New cortical lesions and cortical thickness change after 5 years follow-up of patients with DD <5 years and DD >5 years. The asterisk (*) indicates *p* < 0.001 compared to Patients with DD >5 years (RRMS and SPMS). Regions with more than 0.5% of cortical lesions are shown in the Table.
(PDF)
