# Supplementary material for: Regional Distribution and Evolution of Gray Matter Damage in Different Populations of Multiple Sclerosis Patients
Source: PLoS One. 2015 Aug 12;10(8):e0135428. doi: 10.1371/journal.pone.0135428 (PMC4534410; doi:10.1371/journal.pone.0135428)
Supplement: S1 Table — (PDF) [file pone.0135428.s002.pdf]

	Whole group (n=96)								CIS (n=20)							
	% of new CLs				CTh change (%)				% of new CLs				CTh change (%)			
	Mean	SD	Min	Max	Mean	SD	Min	Max	Mean	SD	Min	Max	Mean	SD	Min	Max
Hippocampal and parahippocampal	**9,1%**	3,2%	4,6%	16,5%	**5,2%**	2,8%	0,5%	11,2%	**12,7%**	2,9%	6,2%	16,5%	**7,7%**	2,0%	2,4%	11,2%
Insular	**8,9%**	3,4%	4,7%	15,4%	**5,4%**	1,8%	0,5%	12,1%	**10,9%**	2,7%	4,8%	14,2%	**6,2%**	1,9%	2,9%	12,1%
Cingulate	**8,3%**	3,6%	4,1%	13,4%	**5,0%**	2,9%	0,6%	11,6%	**11,7%**	3,2%	5,8%	13,4%	**7,0%**	2,4%	1,5%	10,4%
Frontal superior	**8,2%**	4,0%	3,2%	13,5%	**4,5%**	1,6%	0,6%	13,4%	**11,2%**	2,8%	4,2%	13,5%	**7,2%**	1,1%	1,4%	12,2%
Cerebellum	**6,8%**	1,0%	2,8%	12,5%	**5,2%**	1,5%	1,1%	13,3%	**3,9%**	0,6%	2,8%	5,9%	**3,5%**	1,2%	1,1%	6,4%
Precentral	**5,1%**	2,3%	1,1%	12,1%	**3,9%**	1,6%	0,6%	10,8%	**2,6%**	2,6%	1,4%	4,2%	**1,3%**	1,1%	0,8%	6,7%
Frontal middle	**4,9%**	3,1%	1,8%	10,9%	**2,8%**	2,0%	0,8%	14,3%	**5,5%**	2,3%	2,7%	10,9%	**2,3%**	1,7%	1,2%	11,7%
Frontal Inferior	**4,8%**	4,5%	2,8%	12,4%	**2,0%**	4,0%	0,3%	13,6%	**6,1%**	2,1%	3,5%	12,4%	**2,9%**	2,5%	0,6%	6,7%
Parietal superior	**4,3%**	3,2%	1,1%	6,6%	**2,1%**	2,2%	0,8%	11,8%	**3,7%**	3,0%	1,7%	6,4%	**0,9%**	2,1%	0,8%	5,6%
Postcentral	**3,5%**	1,2%	2,0%	6,4%	**4,0%**	2,7%	1,0%	10,0%	**2,2%**	0,7%	2,0%	2,8%	**1,1%**	2,5%	1,0%	4,5%
Precuneus	**3,2%**	1,0%	1,6%	7,1%	**3,8%**	3,4%	0,8%	14,7%	**5,4%**	1,1%	3,0%	7,1%	**1,4%**	2,3%	1,7%	6,5%
Temporal superior	**3,1%**	1,5%	1,0%	5,2%	**4,0%**	4,2%	0,9%	9,6%	**2,9%**	1,0%	1,0%	4,9%	**3,4%**	3,6%	0,9%	6,8%
Paracentral	**2,6%**	0,9%	0,8%	5,2%	**3,5%**	2,0%	0,9%	11,7%	**1,1%**	0,9%	0,8%	3,2%	**2,1%**	1,6%	0,9%	8,4%
Temporal inferior	**2,5%**	1,0%	0,9%	5,2%	**1,6%**	1,6%	0,5%	10,0%	**2,1%**	1,0%	0,9%	2,3%	**1,2%**	1,9%	0,7%	5,7%
Parietal inferior	**2,2%**	1,1%	0,7%	4,1%	**3,5%**	2,1%	1,0%	10,4%	**1,4%**	1,3%	0,7%	3,0%	**1,7%**	2,1%	1,0%	6,7%
Temporal middle	**2,1%**	0,9%	0,9%	3,6%	**2,1%**	5,0%	1,0%	11,4%	**2,0%**	0,5%	1,0%	3,6%	**2,1%**	4,1%	1,2%	8,7%
Cuneus	**2,1%**	0,4%	0,5%	3,5%	**4,5%**	3,3%	1,1%	11,7%	**1,0%**	0,3%	0,5%	2,6%	**2,5%**	3,1%	1,1%	7,6%
Rectus	**2,1%**	1,0%	0,4%	3,2%	**2,6%**	1,6%	0,6%	6,4%	**1,0%**	1,0%	0,6%	1,4%	**1,3%**	1,8%	0,7%	5,2%
Orbital	**1,9%**	1,5%	0,3%	4,8%	**2,7%**	1,9%	0,7%	10,9%	**2,4%**	1,4%	0,8%	4,8%	**3,6%**	1,2%	2,5%	8,2%
Occipital inferior	**1,9%**	0,9%	0,2%	4,2%	**3,5%**	1,8%	0,6%	9,9%	**0,9%**	0,7%	0,3%	2,5%	**1,8%**	1,5%	0,6%	8,4%
Occipital superior	**1,8%**	0,8%	0,3%	4,2%	**3,2%**	1,8%	1,0%	8,2%	**0,8%**	0,5%	0,3%	4,0%	**2,4%**	1,6%	1,0%	6,5%
Calcarine	**1,8%**	0,9%	0,9%	7,4%	**4,9%**	1,7%	1,1%	8,8%	**1,0%**	0,8%	0,9%	1,5%	**3,1%**	1,3%	1,1%	6,4%
Subcentral	**1,8%**	1,1%	0,3%	3,1%	**3,2%**	1,8%	0,7%	10,4%	**1,6%**	1,2%	0,9%	2,4%	**2,5%**	1,4%	0,7%	8,3%
Frontomarginal	**1,6%**	0,4%	1,0%	2,1%	**3,4%**	1,2%	0,6%	9,9%	**1,4%**	0,7%	1,0%	2,1%	**2,1%**	1,0%	0,6%	7,1%
Temporal pole	**1,5%**	1,1%	0,5%	2,1%	**2,0%**	1,5%	0,7%	7,6%	**1,0%**	1,0%	0,5%	1,5%	**1,7%**	1,5%	0,7%	5,6%
Occipital pole	**1,0%**	0,5%	0,3%	1,5%	**3,0%**	1,2%	0,6%	8,4%	**0,9%**	0,6%	0,3%	1,3%	**1,0%**	1,1%	0,6%	6,4%
Frontopolar	**0,8%**	0,5%	0,2%	1,4%	**4,2%**	1,3%	0,6%	7,7%	**0,4%**	0,3%	0,2%	1,4%	**2,4%**	1,6%	0,8%	4,5%
Occipito-temporal	**0,8%**	0,3%	0,1%	1,4%	**2,6%**	1,4%	0,7%	6,9%	**1,3%**	0,3%	0,1%	1,1%	**2,2%**	1,1%	0,8%	4,6%
Lateral fissure	**0,5%**	0,2%	0,0%	1,2%	**3,3%**	2,4%	0,8%	7,9%	**0,5%**	0,2%	0,0%	0,6%	**0,9%**	0,8%	0,8%	5,1%
Occipital middle	**0,5%**	0,2%	0,0%	1,0%	**1,9%**	1,3%	0,5%	6,7%	**0,4%**	0,2%	0,0%	0,6%	**1,5%**	1,0%	0,7%	4,2%

	**early RRMS (n=27)**								**late RRMS (n=29)**							
	**% of new CLs**				**CTh change (%)**				**% of new CLs**				**CTh change (%)**			
	Mean	SD	Min	Max	Mean	SD	Min	Max	Mean	SD	Min	Max	Mean	SD	Min	Max
Hippocampal and parahippocampal	**13,5%**	2,8%	6,9%	14,6%	**5,6%**	2,2%	2,1%	13,3%	**5,4%**	2,1%	4,6%	12,4%	**3,9%**	2,0%	0,5%	7,9%
Insular	**11,4%**	3,0%	4,7%	15,4%	**7,0%**	1,8%	2,8%	13,9%	**6,8%**	2,4%	5,7%	11,2%	**3,6%**	1,4%	0,5%	8,4%
Cingulate	**10,8%**	3,5%	6,1%	12,9%	**6,7%**	2,9%	2,4%	11,6%	**5,7%**	2,3%	4,7%	10,3%	**3,8%**	2,3%	0,6%	10,0%
Frontal superior	**9,3%**	3,8%	4,8%	12,1%	**4,3%**	1,3%	1,6%	13,4%	**6,9%**	3,1%	3,9%	11,8%	**3,8%**	1,2%	0,8%	9,7%
Cerebellum	**4,4%**	0,8%	2,8%	3,2%	**3,0%**	1,1%	2,0%	8,5%	**8,4%**	2,4%	2,8%	7,2%	**6,8%**	2,1%	2,5%	11,4%
Precentral	**2,4%**	2,1%	1,1%	7,1%	**1,0%**	0,9%	0,6%	8,5%	**7,0%**	2,1%	1,1%	12,1%	**6,9%**	1,7%	0,8%	9,3%
Frontal middle	**5,5%**	2,0%	2,7%	10,9%	**3,2%**	2,0%	1,4%	14,3%	**4,4%**	1,4%	1,8%	8,3%	**2,1%**	1,6%	0,8%	9,4%
Frontal Inferior	**4,8%**	2,2%	4,0%	11,5%	**2,0%**	3,2%	1,4%	9,1%	**4,5%**	1,2%	2,8%	10,1%	**1,8%**	1,8%	0,6%	11,2%
Parietal superior	**2,7%**	3,2%	1,7%	5,3%	**1,6%**	1,9%	0,9%	7,9%	**5,9%**	1,4%	1,3%	6,4%	**2,6%**	1,9%	0,8%	9,4%
Postcentral	**2,2%**	0,9%	2,0%	3,9%	**3,2%**	2,1%	1,2%	6,1%	**4,9%**	1,6%	2,0%	5,7%	**6,5%**	2,1%	2,0%	8,6%
Precuneus	**3,5%**	1,0%	3,0%	5,2%	**4,9%**	2,3%	1,6%	7,6%	**2,0%**	0,9%	2,1%	2,8%	**3,5%**	2,3%	0,8%	12,1%
Temporal superior	**2,7%**	1,0%	1,4%	4,5%	**3,4%**	4,0%	0,9%	9,6%	**3,6%**	1,4%	1,0%	5,2%	**4,8%**	2,0%	1,2%	5,9%
Paracentral	**4,3%**	0,9%	1,1%	5,2%	**2,6%**	1,6%	1,4%	10,2%	**2,6%**	0,9%	0,8%	3,0%	**3,2%**	1,5%	1,3%	11,7%
Temporal inferior	**2,6%**	1,0%	0,9%	1,5%	**1,5%**	1,8%	0,5%	7,7%	**2,9%**	1,0%	0,9%	5,2%	**2,0%**	1,8%	0,7%	7,4%
Parietal inferior	**1,6%**	1,1%	0,9%	2,8%	**2,4%**	1,8%	1,4%	8,3%	**2,9%**	0,8%	0,7%	4,1%	**5,3%**	2,4%	1,4%	10,4%
Temporal middle	**2,3%**	0,9%	0,9%	3,6%	**1,9%**	3,9%	1,1%	11,4%	**2,0%**	0,9%	1,0%	3,5%	**2,5%**	3,6%	1,2%	9,3%
Cuneus	**1,2%**	0,4%	0,5%	2,2%	**1,8%**	3,0%	1,2%	9,3%	**3,3%**	0,4%	0,5%	2,6%	**6,2%**	2,1%	1,6%	11,7%
Rectus	**1,0%**	1,0%	0,6%	1,5%	**1,0%**	1,6%	0,6%	6,3%	**3,4%**	1,4%	0,4%	1,5%	**3,1%**	1,3%	1,0%	6,4%
Orbital	**2,5%**	1,5%	2,0%	4,8%	**5,3%**	1,1%	2,3%	10,9%	**1,1%**	1,7%	0,3%	2,5%	**1,4%**	1,1%	0,7%	6,5%
Occipital inferior	**1,2%**	0,9%	0,8%	2,1%	**1,8%**	1,3%	0,6%	9,9%	**2,4%**	0,9%	0,2%	2,5%	**4,2%**	1,1%	1,4%	9,7%
Occipital superior	**0,8%**	0,8%	0,4%	2,3%	**1,9%**	1,6%	1,1%	8,2%	**2,6%**	0,8%	0,3%	4,2%	**4,3%**	1,2%	1,0%	7,5%
Calcarine	**1,0%**	0,8%	0,9%	1,5%	**5,2%**	1,3%	2,1%	8,4%	**2,4%**	0,9%	2,1%	7,4%	**5,1%**	2,1%	1,1%	8,8%
Subcentral	**1,3%**	1,1%	0,9%	2,1%	**2,2%**	1,2%	1,1%	10,4%	**2,2%**	1,0%	0,7%	2,4%	**3,7%**	1,6%	1,7%	10,0%
Frontomarginal	**1,7%**	0,5%	1,3%	2,1%	**4,6%**	1,0%	1,3%	9,9%	**1,6%**	0,5%	1,4%	2,0%	**3,5%**	1,0%	2,0%	7,1%
Temporal pole	**1,0%**	1,0%	0,6%	1,4%	**1,7%**	1,5%	0,7%	6,8%	**2,3%**	1,0%	0,7%	1,5%	**2,3%**	1,3%	1,6%	7,6%
Occipital pole	**1,5%**	0,5%	0,3%	1,2%	**1,0%**	1,0%	0,6%	8,4%	**0,6%**	0,4%	0,4%	1,3%	**4,1%**	0,9%	1,8%	8,3%
Frontopolar	**1,1%**	0,5%	0,4%	1,4%	**3,5%**	1,2%	0,6%	6,8%	**0,7%**	0,8%	0,4%	1,4%	**4,0%**	1,8%	1,3%	7,7%
Occipito-temporal	**0,8%**	0,3%	0,4%	1,1%	**2,5%**	1,1%	0,7%	6,8%	**0,5%**	0,3%	0,4%	1,3%	**2,9%**	1,0%	1,7%	6,9%
Lateral fissure	**0,5%**	0,2%	0,2%	0,6%	**2,5%**	1,2%	1,1%	7,9%	**0,4%**	0,1%	0,3%	1,0%	**4,1%**	2,0%	1,0%	5,4%
Occipital middle	**0,4%**	0,2%	0,2%	0,6%	**1,5%**	1,0%	0,5%	5,8%	**0,6%**	0,2%	0,4%	0,9%	**2,7%**	1,0%	1,7%	6,7%

	SPMS (n=20)							
	% of new CLs				CTh change (%)			
	Mean	SD	Min	Max	Mean	SD	Min	Max
Hippocampal and parahippocampal	**5,1%**	2,5%	5,0%	11,2%	**3,7%**	2,1%	0,4%	10,5%
Insular	**6,6%**	2,7%	5,9%	10,4%	**4,0%**	1,8%	0,5%	10,7%
Cingulate	**5,4%**	3,6%	4,1%	9,2%	**3,0%**	2,5%	1,3%	11,3%
Frontal superior	**5,6%**	3,2%	3,2%	11,8%	**2,8%**	1,4%	0,6%	11,4%
Cerebellum	**10,4%**	0,8%	4,2%	12,5%	**6,5%**	1,0%	3,0%	13,3%
Precentral	**8,5%**	2,6%	5,0%	12,0%	**6,1%**	0,9%	2,3%	10,8%
Frontal middle	**4,4%**	2,0%	4,0%	6,4%	**3,3%**	1,6%	1,1%	11,0%
Frontal Inferior	**4,1%**	2,1%	2,8%	8,3%	**1,1%**	3,2%	0,3%	13,6%
Parietal superior	**4,8%**	3,0%	1,1%	6,6%	**3,1%**	1,9%	1,2%	11,8%
Postcentral	**4,7%**	0,9%	2,0%	6,4%	**6,5%**	2,0%	2,7%	10,0%
Precuneus	**2,2%**	0,9%	1,6%	2,8%	**4,0%**	2,3%	1,3%	14,7%
Temporal superior	**3,0%**	1,0%	1,0%	4,9%	**4,3%**	3,2%	1,1%	7,8%
Paracentral	**2,0%**	0,9%	0,8%	3,0%	**5,4%**	1,5%	2,1%	10,5%
Temporal inferior	**2,4%**	1,0%	0,9%	5,2%	**1,6%**	1,8%	0,5%	10,0%
Parietal inferior	**2,8%**	1,1%	1,0%	4,1%	**4,4%**	1,8%	2,4%	8,4%
Temporal middle	**2,3%**	0,9%	1,1%	3,5%	**2,0%**	3,6%	1,0%	7,9%
Cuneus	**2,9%**	0,4%	0,7%	3,5%	**7,2%**	2,5%	3,0%	10,6%
Rectus	**2,6%**	1,0%	0,8%	3,2%	**4,7%**	1,6%	3,1%	4,9%
Orbital	**1,6%**	1,4%	0,5%	2,6%	**1,4%**	1,1%	0,7%	5,1%
Occipital inferior	**2,9%**	0,9%	1,1%	4,2%	**6,0%**	1,1%	1,6%	7,7%
Occipital superior	**3,2%**	0,8%	1,0%	4,2%	**4,3%**	1,6%	2,3%	5,7%
Calcarine	**2,9%**	0,8%	2,1%	7,4%	**7,3%**	0,9%	2,0%	7,5%
Subcentral	**2,0%**	1,1%	0,3%	3,1%	**4,3%**	1,5%	1,9%	9,0%
Frontomarginal	**1,6%**	0,5%	1,1%	2,0%	**3,5%**	1,0%	1,4%	5,9%
Temporal pole	**1,7%**	1,0%	0,9%	2,1%	**2,3%**	1,3%	1,1%	6,6%
Occipital pole	**1,1%**	0,4%	0,4%	1,5%	**5,5%**	0,9%	1,9%	6,9%
Frontopolar	**1,1%**	0,6%	0,4%	1,4%	**6,9%**	1,2%	2,3%	6,7%
Occipito-temporal	**0,9%**	0,3%	0,4%	1,4%	**2,9%**	1,0%	1,3%	4,9%
Lateral fissure	**0,6%**	0,2%	0,2%	1,2%	**5,2%**	1,3%	1,5%	3,8%
Occipital middle	**0,6%**	0,2%	0,5%	1,0%	**2,0%**	1,0%	1,2%	5,4%
