# Supplementary material for: Regional Distribution and Evolution of Gray Matter Damage in Different Populations of Multiple Sclerosis Patients
Source: PLoS One. 2015 Aug 12;10(8):e0135428. doi: 10.1371/journal.pone.0135428 (PMC4534410; doi:10.1371/journal.pone.0135428)
Supplement: S2 Table — The asterisk (*) indicates p < 0.001 compared to Patients with DD >5 years (RRMS and SPMS). Regions with more than 0.5% of cortical lesions are shown in the Table. (PDF) [file pone.0135428.s003.pdf]

	Patients with DD <5 years (CIS and eaarly RRMS) (n=47)								Patients with DD >5 years (RRMS and SPMS) (n=49)							
	% of new CLs				CTh change (%)				% of new CLs				CTh change (%)			
	Mean	SD	Min	Max	Mean	SD	Min	Max	Mean	SD	Min	Max	Mean	SD	Min	Max
Whole brain	100,0%				2,8%	1,8%	0,6%	12,2%	100,0%				4,0%	1,7%	0,5%	12,1%
Hippocampal and parahippocampal	13,2%*	2,8%	6,2%	16,5%	6,7%*	2,2%	2,4%	11,2%	5,2%	2,5%	4,6%	12,4%	3,8%	2,1%	0,5%	7,9%
Insular	11,2%*	3,0%	4,7%	15,4%	7,1%*	1,8%	2,9%	12,1%	6,7%	2,7%	4,7%	14,3%	3,8%	1,8%	0,5%	8,4%
Frontal superior	10,1%*	3,8%	4,2%	13,5%	5,9%*	1,3%	1,4%	12,2%	6,2%	3,2%	3,9%	11,8%	3,2%	1,4%	0,8%	9,7%
Cingulate	11,2%*	3,5%	5,8%	13,4%	6,8%*	2,9%	1,5%	10,4%	5,5%	3,6%	4,7%	10,3%	3,3%	2,5%	0,6%	10,0%
Cerebellum	3,3%*	0,8%	2,8%	5,9%	3,2%*	1,1%	1,1%	6,4%	9,6%	0,8%	2,8%	7,2%	7,1%	1,0%	2,5%	11,4%
Frontal Inferior	7,1%*	2,2%	3,5%	12,4%	2,6%	3,2%	0,6%	6,7%	4,3%	2,1%	2,8%	10,1%	1,4%	3,2%	0,6%	11,2%
Frontal middle	6,1%*	2,0%	2,7%	10,9%	2,8%	2,0%	1,2%	11,7%	4,4%	2,0%	1,8%	8,3%	2,8%	1,6%	0,8%	9,4%
Precentral	2%*	2,1%	1,1%	7,1%	1,1%*	0,9%	0,8%	6,7%	7,9%	2,6%	1,1%	12,1%	6,6%	0,9%	0,8%	9,3%
Parietal superior	3,1%*	3,2%	1,3%	6,4%	1,3%*	1,9%	0,8%	5,6%	5,3%	3,0%	1,3%	6,4%	2,9%	1,9%	0,8%	9,4%
Postcentral	2,1%*	0,9%	2,3%	5,1%	1,3%*	2,1%	1,0%	4,5%	4,8%	0,9%	2,0%	5,7%	6,6%	2,0%	2,0%	8,6%
Precuneus	4,9%*	1,0%	3,0%	5,2%	3,8%	2,3%	1,7%	6,5%	2,1%	0,9%	2,1%	2,8%	3,8%	2,3%	0,8%	12,1%
Temporal superior	2,8%	1,0%	1,0%	4,9%	3,4%	4,0%	0,9%	6,8%	3,3%	1,0%	1,0%	5,2%	4,5%	3,2%	1,2%	5,9%
Temporal inferior	2,4%	1,0%	0,9%	5,2%	1,4%	1,8%	0,7%	5,7%	2,6%	1,0%	0,9%	5,2%	1,8%	1,8%	0,7%	7,4%
Temporal middle	2,2%	0,9%	1,0%	3,6%	2,0%	3,9%	1,2%	8,7%	2,2%	0,9%	1,0%	3,5%	2,2%	3,6%	1,2%	9,3%
Orbital	3%*	1,5%	0,8%	4,8%	4,1%*	1,1%	2,5%	8,2%	1,4%	1,4%	0,3%	2,5%	1,4%	1,1%	0,7%	6,5%
Parietal inferior	1,5%*	1,1%	0,7%	3,0%	2,1%*	1,8%	1,0%	6,7%	2,8%	1,1%	0,7%	4,1%	4,8%	1,8%	1,4%	10,4%
Cuneus	1,1%*	0,4%	0,5%	2,6%	2,1%*	3,0%	1,1%	7,6%	3,1%	0,4%	0,5%	2,6%	6,8%	2,5%	1,6%	11,7%
Rectus	1%*	1,0%	0,6%	1,5%	1,1%*	1,6%	0,7%	5,2%	3,0%	1,0%	0,4%	1,5%	4,0%	1,6%	1,0%	6,4%
Occipital superior	0,8%*	0,8%	0,3%	4,0%	2,1%*	1,6%	1,0%	6,5%	3,0%	0,8%	0,3%	4,2%	4,3%	1,6%	1,0%	7,5%
Occipital inferior	1,1%*	0,9%	0,3%	2,5%	1,8%*	1,3%	0,6%	8,4%	2,7%	0,9%	0,2%	2,5%	5,2%	1,1%	1,4%	9,7%
Paracentral	1,2%*	0,9%	0,8%	3,2%	2,4%*	1,6%	0,9%	8,4%	2,3%	0,9%	0,8%	3,0%	4,5%	1,5%	1,3%	11,7%
Subcentral	1,4%	1,1%	0,9%	2,4%	2,3%*	1,2%	0,7%	8,3%	2,1%	1,1%	0,7%	2,4%	4,1%	1,5%	1,7%	10,0%
Frontomarginal	1,6%	0,5%	1,4%	2,1%	3,4%	1,0%	0,6%	7,1%	1,6%	0,5%	1,4%	2,0%	3,5%	1,0%	2,0%	7,1%
Calcarine	1%*	0,8%	0,9%	1,5%	3,4%*	1,3%	1,1%	6,4%	2,7%	0,8%	2,1%	7,4%	6,3%	0,9%	1,1%	8,8%
Temporal pole	1%*	1,0%	0,5%	1,5%	1,7%	1,5%	0,7%	5,6%	2,0%	1,0%	0,7%	1,5%	2,3%	1,3%	1,6%	7,6%
Frontopolar	0,8%	0,5%	0,4%	1,4%	2,7%*	1,2%	0,8%	4,5%	0,9%	0,6%	0,4%	1,4%	5,6%	1,2%	1,3%	7,7%
Occipito-temporal	1,0%	0,3%	0,3%	1,1%	2,4%	1,1%	0,8%	4,6%	0,7%	0,3%	0,4%	1,3%	2,9%	1,0%	1,7%	6,9%
Occipital pole	0,9%	0,5%	0,3%	1,3%	1%*	1,0%	0,6%	6,4%	0,9%	0,4%	0,4%	1,3%	4,9%	0,9%	1,8%	8,3%

Lateral fissure	0,5%	0,2%	0,2%	0,6%	1,8%*	1,2%	0,8%	5,1%	0,5%	0,2%	0,3%	1,0%	4,7%	1,3%	1,0%	5,4%
Occipital middle	0,4%	0,2%	0,2%	0,6%	1,5%	1,0%	0,7%	4,2%	0,6%	0,2%	0,4%	0,9%	2,3%	1,0%	1,7%	6,7%
